# Protective Effect of HLA-B*5701 and HLA-C -35 Genetic Variants in HIV-Positive Caucasians from Northern Poland

**DOI:** 10.1371/journal.pone.0127867

**Published:** 2015-06-11

**Authors:** Magdalena Leszczyszyn-Pynka, Bogusz Aksak-Wąs, Anna Urbańska, Miłosz Parczewski

**Affiliations:** Department of Infectious, Tropical Diseases and Acquired Immunodeficiency, Pomeranian Medical University, Szczecin, Poland; Harvard Medical School, UNITED STATES

## Abstract

**Aim of the Study:**

Association of two HLA class I variants with HIV-1 pretreatment viremia, CD4+ T cell count at the care-entry and CD4+ T cell nadir.

**Methods:**

414 HIV-positive Caucasians (30% women) aged 19-73 years were genotyped for HLA-C -35 (rs9264942) and HLA-B*5701 variants. HIV-1 viral load, as well as CD4+ T cell count at care-entry and nadir, were compared across alleles, genotypes and haplotypes.

**Results:**

HLA-C -35 C/C genotype was found in 17.6% patients, C/T genotype in 48.1%, and T/T genotype in 34.3% patients. HLA-B*5701 variant was present in 5.8% of studied population. HIV plasma viremia in the group with C allele was significantly lower (p=0.0002) compared to T/T group [mean:4.66 log (SD:1.03) vs. 5.07 (SD:0.85) log HIV-RNA copies/ml, respectively], while CD4+ T cell count at baseline was notably higher among C allele carriers compared to T/T homozygotes [median: 318 (IQR:127-537) cells/μl vs. median: 203 (IQR:55-410) cells/μl, respectively] (p=0.0007). Moreover, CD4+ T cell nadir among patients with C allele [median: 205 (IQR:83.5-390) cells/μl] was significantly higher compared to T/T group [median: 133 (IQR:46-328) cells/μl] (p=0.006). Among cases with HLA-B*5701 allele, significantly lower pretreatment viremia and higher baseline CD4+ T cell count were found (mean: 4.08 [SD: 1.2] vs. mean: 4.84 [SD:0.97] log HIV-RNA copies/ml, p=0.003 and 431 vs. 270 cells/μl, p=0.04, respectively) compared to HLA-B*5701 negative individuals. The lowest viremia (mean: 3.85 log [SD:1.3]) HIV-RNA copies/ml and the highest baseline and nadir CD4+ T cell [median: 476 (IQR:304-682) vs. median: 361 (IQR: 205-574) cells/μl, respectively) were found in individuals with HLA-B*5701(+)/HLA-C –35 C/C haplotype.

**Conclusions:**

HLA-C -35 C and HLA-B*5701 allele exert a favorable effect on the immunological (higher baseline and nadir CD4+ T cell count) and virologic (lower pretreatment HIV viral load) variables. This protective effect is additive for the compound HLA-B*5701(+)/HLA-C -35 C/C haplotype.

## Introduction

Human leukocyte antigens C (HLA-C) strongly influence immunological activity in chronic viral infections (HCV, HIV) as well as in autoimmune diseases e.g. Crohn’s disease, autoimmune liver diseases, Graves’ disease and psoriasis vulgaris [1,2,3,4,5].

HLA-C antigens play an important role in HIV control through two mechanisms: acting as ligands for killer immunoglobulin-like receptors (KIRs) presented on natural killer (NK) cells and directly by antigen (e.g. viral) presentation to cytotoxic T cells [1,6,7].

The degree of NK cell activation or inhibition depends, *inter alia*, on the level of HLA-C expression [8]. HLA-C single nucleotide polymorphism (SNP) rs9264942 in locus -35 was found to be associated with differences in HIV-1 viremia in antiretroviral naive individuals [4,7,9,10,11,12,13,14]. The mechanism of this phenomenon is most likely related to differences in the HLA-C mRNA and surface expression levels between the -35 C and T allele carriers [9]. Among the -35 C/C homozygotes, overall HLA-C expression levels were approximately 1.7 fold higher compared to the cases with -35 T/T genotype [9]. It should be noted that -35 C allele tends to be in linkage disequilibrium with the protective HLA-B*57 and HLA-B*27, while the -35 T allele tends to be linked with the HLA-Cw7 allele, which has been associated with more rapid progression of the disease [15]. HLA-Cw7 is known to be a contributor to the low HLA-C expression on the cell surface and is associated with progression to advanced disease [9]. Being a strong ligand for KIR receptors on NK cells, HLA-C protects target cells (e.g. neoplastic or infected with a virus) from NK-mediated lysis [16]. However, HLA-C is much less efficient in presenting antigen to cytotoxic T lymphocytes than HLA-A and HLA-B [17].

Host genetics related control and virological outcomes of HIV infection are also known to be associated with HLA-B*5701 and are likely to be related to the induction of the effective T cell response and variability of the peptide binding groove [11,13, 18].

The array of HLA genetic variants affects not only the HIV-1 viremia but also the longitudinal viral load levels, as well as outcomes of HIV infection such as time from acute retroviral syndrome to CD4+ T cells decrease ≤ 200 cells/μl. HLA-B*5701 and HLA-C locus -35 (rs9264942) C allele were clearly defined as protective against progression of the HIV disease [8, 19, 20]. Moreover, the frequency of protective HLA-B*5701 and HLA-C locus -35 C/C homozygotes was higher among individuals with natural HIV control defined as the ability to maintain the plasma viremia below 2000 copies/ml [21].

In this study, we aimed to investigate the association between two genetic variants, namely HLA-C -35 (rs9264942) and HLA-B*5701, pretreatment HIV-1 viral load and CD4+ T cell count as well as CD4+ T cell nadir. Additionally, we wished to investigate the effect of compound HLA-C -35/HLA-B*5701 haplotype on the aforementioned characteristics.

## Methods

### Study group

For the study, data and samples from 414 HIV-1 positive patients followed-up from January 2000 to April 2014 at the Department of Infectious, Tropical Diseases and Immunodeficiency, Pomeranian Medical University, and Out Patients’ Clinic for Acquired Immunodeficiency, Regional Hospital, Szczecin, Poland were analyzed. The study protocol was approved by the institutional review board—the Bioethical Committee of Pomeranian Medical University in Szczecin (Komisja Bioetyczna Pomorskiego Uniwersytetu Medycznego w Szczecinie), Poland (approval number KB-0012/87/10). Written informed consent, approved by the Bioethical Committee, was obtained from the study participants and recorded in the study files.

Full blood samples were used for DNA extraction and HLA genotyping. The following clinical data were collected: age, gender, route of transmission, hepatitis C co-infection, clinical category at diagnosis according to Centers’ for Disease Control (CDC, USA) case definition, pretreatment HIV viral load, baseline and nadir lymphocyte CD4 counts. Baseline CD4+ T cell counts were defined as the first documented result after diagnosis of HIV. CD4+ T cell nadir was analyzed as a continuous variable. CDC category at diagnosis was defined based on the review of the patient’s clinical record. The case of the late care entry category A (asymptomatic) was assumed if no apparent immunodeficiency was reported or available from the medical record. Data on chronic hepatitis B were not included into the analysis due to a small number of confirmed HIV/HBV co-infection cases.

### HLA genotyping

QIAamp DNA Blood Mini Kit (QIAgen, Hilden, Germany) was used to extract genomic DNA from samples previously collected into tubes containing EDTA anticoagulant. The extraction was performed following manufacturer’s protocol DNA was re-suspended in 200 μL of AE buffer (QIAgen, Hilden, Germany) and stored at 4°C for further analyses. To assess the distribution of HLA-C -35 single nucleotide polymorphism rs9264942, TaqMan SNP (Life Technologies, USA) genotyping assay was used, according to the manufacturer's protocol using real-time PCR technology on the StepOne thermal cycler (Applied Biosystems/Life Technologies, Foster City, CA). Genotypes were identified using TaqMan Genotyper Software v1.0.1 (Applied Biosystems/Life Technologies, Foster City, CA) and calculation of Hardy-Weinberg equilibrium for each analyzed set of genotypes was performed.

HLA-B*5701 screening was performed using SSP HLA-Ready gene B5/57 cross low resolution kit (Inno-Train Diagnostik, Kronberg, Germany) by a diagnostically, *in vitro* validated CE marked test, according to the manufacturer’s protocol. PCR products were electrophoresised on a 3%, agarose gel (SIGMA, Saint Louis, USA) stained with Gel-Star dye (Lonza, Switzerland). Results were visualized under UV light (Transilluminator 4000, Stratagene, La Jolla, USA) and recorded with DS-34 Polaroid Direct Screen Camera. Additionally, all B*5701 positive samples were verified using a CE marked assay, Olerup SSP HLA-B*57 high resolution kit (Olerup SSP AB, Saltsjoebaden, Sweden), with subsequent electrophoresis and recording as described above.

### Statistics

Statistical comparisons were performed using the Fisher’s exact and Chi^2^ tests for nominal variables. Continuous variables were analyzed using the t-test for normally distributed ones (HIV-1 viral load), while U-Mann Whitney and ANOVA tests were used for non-parametric statistics (age at diagnosis, baseline and nadir lymphocyte CD4 counts). Linear regression was used to compare continuous variables across genotypes and haplotypes. Statistica 8.0PL software (Statasoft, Poland) was used for statistical calculations.

## Results

Epidemiological, clinical and basic laboratory data are presented in Table 1. No differences neither in the baseline, nadir CD4+ T cell count, nor pretreatment HIV viral load between the male and female gender were found.

**Table 1:** Characteristics of the study participants.

**No of participants** (number of women, %)	414 (124, 30)
**Age**, range in years (median)	19–76 (40)
**Transmission route**	
Intravenous drug use, n (%)	134 (32.4)
Sexual contact, n (%)	270 (65.2)
MSM contact, n (%)	124 (30)
Unknown, n (%)	10 (2.4)
**HCV-positive**, n (%)	220 (53.3%)
**Lymphocyte CD4^+^ T cell count at baseline** ^#^	
range (cells/μl)	0–1707
median (IQR) (cells/μl)	280.5 (94.0–486)
**Nadir lymphocyte CD4^+^ T cell count** ^$^	
range (cells/μl)	0–1189
median (IQR) (cells/μl)	180 (63.5–371)
**HIV viral load at baseline** *	
range log copies/ml	1.4–7.4
mean log copies/ml (SD)	4.8 (0.99)
**HIV infection stage at genotyping according to CDC**, n (%)	
A	140 (33.8)
B	138 (33.3)
C	124 (30)
no data	12 (2.9)
**HIV-1 subtype B** ^@^, n (%)	264 (74.4)
**HIV-1 non-B variants** ^@^, n (%)	91 (25.6)

^#^ available for 408 cases.

^$^available for 384 cases.

*available for 414 cases.

^@^ subtype available for 355 cases.

C/C genotype -35 HLA-C was found in 73 (17.6%) patients, C/T in 199 (48.1% while T/T variant in 142 (34.3%) cases. Allele HLA-B*5701 was present in 24 (5.8%) patients.

**Fig 1:**
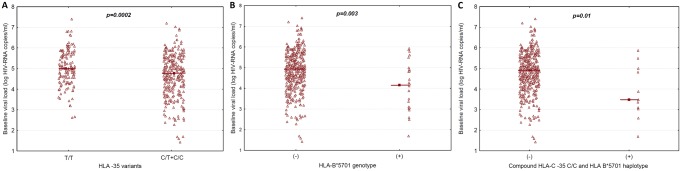
The relationship between pretreatment HIV plasma viremia and (a) HLA-C locus -35 genotypes for T/T homozygotes vs. C allele carriers (C/T+C/C), (b) HLA B*5701(+) vs HLA B*5701(-) genotype, and (c) compound HLA-C locus -35 C/C and HLA B*5701 (+) haplotype. Raw values for each genotype are presented as triangles, means as squares. For statistics t-test was used.

Results of statistical relationships between HLA-B*5701 and HLA-C -35 rs9264942 genetic variants and selected virologic as well as immunologic parameters are shown in Table 2. No gender related differences in the distribution of the HLA-C alleles and genotypes were observed with genotype frequencies of 18.6% (n = 54) for C/C, 47.9% (n = 139) for C/T and 33.5% (n = 97) for T/T for male cases versus 15.3% (n = 19) for C/C, 48.4% (n = 60) for C/T and 36.35% (n = 45) for T/T among women. HLA B*5701 allele frequency was slightly higher among women (11 cases, 8.9%) compared to men (13 cases, 4.48%), p = 0.08. In the group with HLA-C -35 C allele, baseline viral load was significantly lower compared to baseline viremia for T/T homozygotes [mean: 4.66 (SD: 1.03) log HIV-RNA copies/ml vs. mean: 5.07 (SD: 0.85) log HIV-RNA copies/ml (p = 0.0002)] (Fig 1a).

**Table 2:** Association between HLA–C gene variants and selected immunological and virologic data.

Genotype	N (%)	Baseline CD4^+^ T cell count (cells/μl), median (IQR)	p value	Nadir CD4^+^ T cell count (cells/μl), median (IQR)	p value	Baseline HIV viral load, mean log copies/ml (SD)	p value
**HLA-C CC vs. CT vs. TT** ^#^							
CC	73 (17.6)	325 (168–487)	**0.0087**	231 (82–377)	**0.035**	4.59 (1.11)	**0.002**
CT	199 (48.1)	317 (119–542)		187 (88–393)		4.68 (1.01)	
TT	142 (34.3)	202.5 (55–410)		133 (46–328)		5.07 (0.85)	
**HLA-C CC vs. CT + TT** ^§^							
CC	73 (17.6)	325 (168–487)	0.12	231 (82–377)	0.13	4.59 (1.11)	0.11
CT +TT	341 (82.4)	268 (81–485)		171 (63–365)		4.84 (0.97)	
**HLA-C TT vs. CT + CC** ^§^							
TT	142 (34.3)	202.5 (55–410)	**0.0007**	133 (46–328)	**0.006**	5.07 (0.85)	**0.0002**
CT + CC	272 (65.7)	318 (127–537)		205 (83.5–390)		4.66 (1.03)	
**HLA-B 57** ^§^							
B 57 (+)	24 (5.8)	431 (220–577)	**0.04**	304 (106–521)	0.051	4.08 (1.20)	**0.003**
B 57(-)	390 (94.2)	270 (93.5–477.5)		176 (61–363)		4.84 (0.97)	
**HLA- B 57(+) + HLA-C CC vs. HLA- B 57(+) + HLA-C CT + TT + HLA-B 57 (-)** ^§^							
HLA- B 57(+) + HLA-C CC	11 (2.7)	476 (304–682)	**0.04**	361 (205–574)	**0.026**	3.85 (1.3)	**0.01**
HLA-B 57(+) + HLA-C CT + TT + HLA-B 57(-)	403 (97.3)	277 (94–482)		176 (63–365)		4.82 (0.98)	

^#^ Calculated with ANOVA test.

^§^ Calculated with Chi^2^ test.

**Fig 2:**
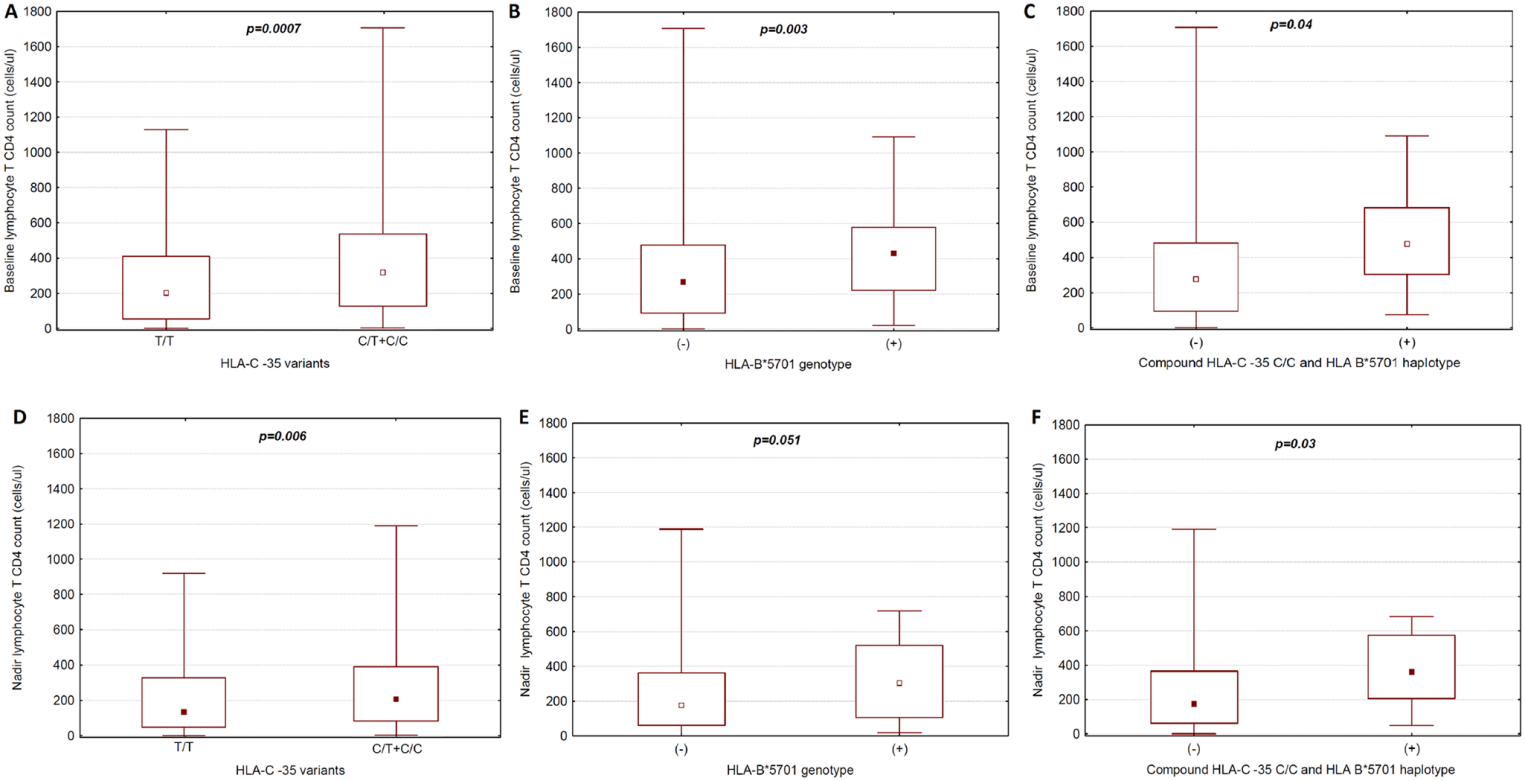
Box-whisker plot of the relationship between baseline lymphocyte CD 4 cell count and (a) HLA-C locus -35 genotypes for T/T homozygotes vs. C allele carriers (C/T+C/C), (b) HLA B*5701 genotype, (c) compound HLA-C locus -35 C/C and HLA B*5701 haplotype as well as nadir lymphocyte CD 4 cell count and (d) HLA-C locus -35 genotypes for T/T homozygotes vs. C allele carriers (C/T+C/C), (e) HLA B*5701 (+) genotype, (f) compound HLA-C locus -35 C/C and HLA B*5701(+) haplotype. Boxes are IQR. Whiskers are CD4 count ranges. Central square inside a box represents a median. For statistics U-Mann-Whitney test was implemented.

Also, baseline CD4+ T cell count among HLA-C -35 C allele carriers was significantly higher compared to T/T group [median: 318 (IQR: 127–537) cells/μl vs. 210 (IQR: 55–410) cells/μl] (p = 0.0007) (Fig 2a).

Similarly, the CD4+ T cell nadir among patients with at least one HLA-C -35 C allele was significantly higher compared to T/T homozygotes [median: 205 (IQR: 84–390) cells/μl vs. median: 133 (IQR: 46–328) cells/μl] (p = 0.006) (Fig 2d).

In patients with HLA-B*5701variant, significantly lower pretreatment viral load and higher CD4+ T cell count at baseline were observed in comparison with HLA B*5701 negative group [mean: 4,08 (SD: 1,2) vs. mean: 4.84 (SD: 0.97) log HIV-RNA copies/ml, p = 0.003 and median: 431 vs. median: 270 cells/μl, p = 0.04) (Figs 1b and 2b). Differences for the CD4+ T cell count between the HLA B*5701 positive and negative individuals were of the borderline statistical significance (p = 0.051) (Fig 2e).

The lowest pretreatment viremia (mean: 3.85 log HIV-RNA copies/ml, SD: 1.3), the highest baseline and nadir CD4+ T cell count [median: 476 (IQR: 304–682) and median: 361 (IQR: 205–574) cells/μl, respectively] were found in the group with the compound HLA-B*5701(+)/HLA-C locus -35 C/C haplotype (Figs 1c, 2c and 2f).

Associations between HLA-B*5701 and HLA-C -35 rs9264942 variants, HIV-1 viral load, baseline CD4+ T cell count and nadir were also analyzed separately for the HIV/HCV coinfected and HIV monoinfected groups. Results were in line with the findings presented for the entire group. In the HIV/HCV coinfected patients, lower pretreatment viral loads were observed for the HLA-C -35 C allele compared to T/T homozygotes [mean: 4.4 (SD: 1.0) vs. mean: 4.86 (SD: 0.85) log HIV-RNA copies/ml for the T/T] (p = 0.04), and for the HLA-B*5701 (+) [mean: 3.4 (SD: 0.49)] compared to the HLA-B*5701 (-) cases [mean: 4.6 (SD: 0.95)]. In patients without HCV coinfection, HLA-B*5701 (+) genotype was associated with higher baseline CD4+ T cell counts [median: 537 (IQR: 361–682) cells/μl] vs. median 208 (IQR: 61–500) cells/μl for the HLA-B*5701 (-) (p = 0.012), higher CD4+ T cell nadir [median: 437 (IQR: 313–628) cells/μl] vs. median 162 (IQR: 45–386) cells/μl (p = 0.08) and lower pretreatment viral loads (mean: 3.87 [SD: 1.3] vs. mean: 4.97 [SD: 0.91] log HIV-RNA copies/ml (p = 0.004). The remaining associations were not significant when analyzed separately for HIV/HCV coinfected and HIV monoinfected subgroups.

**Fig 3:**
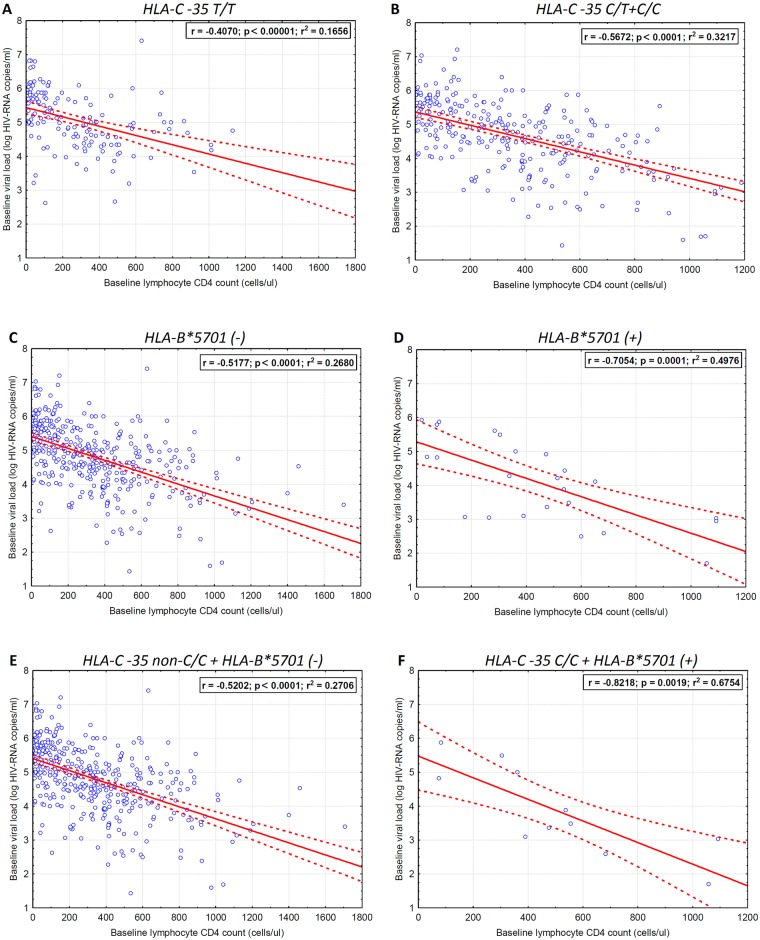
Linear regression curves for the pretreatment HIV-1 viral load data versus baseline lymphocyte CD 4 cell count for (a) HLA-C locus -35 T/T homozygotes, (b) HLA-C locus -35 C allele carriers (C/T+C/C), (c) HLA B*5701 (-) genotype, (d) HLA B*5701 (+) genotype, (e) compound HLA-C locus -35 non-C/HLA B*5701 (-) and (f) HLA-C locus -35 C/C and HLA B*5701(+) haplotype. Dotted line indicates 95 confidence interval for the logistic regression.

Strength of association between baseline viral load and CD4+ T cell count differed across the HLA-C locus -35 groups, with the correlation coefficient being the higher for the group C allele (r^2^ = 0.321, p<0.0001) than among T/T homozygotes (r^2^ = 0.166, p<0.0001) (Fig 3a and 3b), which indicates that T/T homozygotes present with higher viral load regardless the CD4+ T cell count. Similarly, in HLA-B*5701(+) cases the correlation coefficient was higher compared to patients without this allele [r^2^ = 0.498, p = 0.0001 for HLA-B*5701(+) versus r^2^ = 0.268, p<0.0001 for HLA-B*5701(-)] (Fig 3c and 3d). The strongest correlation was found for the HLA-B*5701(+)/HLA-C locus -35 C/C haplotype (r^2^ = 0.676, p = 0.0019) (Fig 3e and 3f).

## Discussion

Favorable effects of certain HLA class I alleles on the outcomes of HIV infection have been shown in many studies [8,11,20, 22, 23]. Functional studies indicate that innate immune response, via NK cells differential activity as well as an acquired immunity response by presentation of HIV epitopes to effector T cells, are involved in an interplay with some HLA variants. The key finding of our study was the confirmation of a favorable effect of both analyzed HLA-C and HLA-B loci on virologic and immunologic characteristics, including the strongest influence of the compound HLA-B*5701/HLA-C -35 C/C haplotype. The population of this study was ethnically homogenous, Caucasian group. Certain differences in the influence of genetic variants and their combinations are dependent on the origin of the subpopulations: Afro-Americans, Euro-Americans, Chinese patients, African groups and multiethnic and were described in terms of immunogenetics and HIV infection outcomes [7,8, 20, 24, 25, 26, 27]. In the Chinese cohort infected with homogenous HIV subtype the protective effect of HLA-C restricted immune response was found; in this study it was confirmed that the strength of immune pressure on HIV is greater among individuals with protective HLA-C genotypes [7]. These results were in line with the genome-wide association study (GWAS) results in population of European ancestry identifying HLA-B and HLA-C variants associated with immunologic and virologic HIV control [11]. Of note, the protective effect found in GWAS for the rs2395029G allele at the *HCP5* locus favorable for the viremia set-point in Caucasians, was not confirmed for Afro-American population [24].

Moreover, some studies were conducted in the populations infected with divergent HIV subtypes [7,25]. Our study included mostly HIV-1 subtype B infected patients; however a subset of non-B variants was also present. Epitope specificity may differ across HIV subtypes and may influence the interplay between the virus and the host, but the exact nature of the interplay of the human HLA variability and HIV subtypes remains to be elucidated.

We found that the -35 SNP rs9264942 allele C is the strongest marker for the lower baseline HIV plasma viremia as well as higher CD4+ T cell count and CD4+ T cell nadirs. It is consistent with the previous studies presenting advantageous influence of this polymorphism [4,7,8,12,13, 22, 28]. Additionally, we confirmed favorable influence of the HLA-B*5701 variant on the baseline CD4+ T cell count and HIV viral load at the care-entry. This genetic variant is well documented and one of the most important factor to mediate HIV-1 infection control [19,20, 23, 25, 26, 29, 30].

The novel finding of this report includes the observation that the HLA-B*5701(+)/HLA-C rs9264942 C/C haplotype is associated with the most favorable pattern of influence on HIV viremia and CD4+ T cell count among the analyzed variants. This haplotype is clearly linked with the lowest pretreatment plasma viral loads, the highest CD4+ T cell counts at care-entry, as well as with the highest nadir of CD4+ T cell count, and reflects delayed progression to immunodeficiency.

We found that the coexistence of two protective HLA gene variants results in an additive protective effect on HIV infection. Some studies revealed that HLA-C variants independently control the HIV infection [8,12,13, 22], while the co-operation of HLA class I genes in terms of viral control was found in the big cohort from South Africa [27].

The protective host genes exert immune pressure on the virus, which creates “escape” mutations. Such finding connected with C/C variant of rs9264942 was presented by Blais and colleagues [7]. The frequency of the alleles in our population was 17.6% which means that such a scenario of rising mutants of HIV may be not uncommon and raises an important issue for the further study. It is likely, that HLA-C -35 C allele may be associated with the differences in the cytotoxic T lymphocyte responses resulting in suppression of HIV replication and evolution of the escape mutations as shown by Honda et al., for the HLA-Cw*1202 restricted pol epitopes. Interestingly, these epitopes remain in linkage disequilibrium with the -35 C/C genotype [31]. HIV-1 inhibitory activity on HLA-C is weak, so the sufficient suppression of NK activity is preserved, giving the advantage for the virus [16]. However, in our study we did not analyze the cytotoxic T lymphocyte cell responses nor NK lytic activity across investigated genotypes.

The limitation of the study is related to the fact, that it was impossible to assess the time from infection to the care entry, therefore differences in HIV plasma viremia and CD4+ T cell counts related to the time of infection in association with investigated HLA variants were not analyzed.

To conclude, we have confirmed the protective effect during the HIV infection of the analyzed HLA variants, including additive one for the compound haplotype. Analysis of genetic variants across various ethnic groups allows confirming the consistence of the genetic effect exerted by the investigated variants.
